# Human Rights To *In Vitro* Fertilization

**DOI:** 10.5935/1518-0557.20140089

**Published:** 2014

**Authors:** Fernando Zegers-Hochschild, Bernard M. Dickens, Sandra Dughman-Manzur

**Affiliations:** 1 Program of Ethics and Public Policies in Human Reproduction, University Diego Portales, Santiago, Chile; 2 Faculty of Law, Faculty of Medicine, and Joint Centre for Bioethics, University of Toronto, Toronto, Canada; 3 Fellow and Consultant, International Reproductive and Sexual Health Law Program, University of Toronto, Toronto, Canada

**Keywords:** Conception, Costa Rica, Fertilization, Human rights to IVF, Infertility, Inter-American Court of Human Rights, *In vitro* fertilization (IVF)

## Abstract

The Inter-American Court of Human Rights has ruled that the Supreme Court of Costa Rica’s 2000 judgment prohibiting *in vitro* fertilization (IVF) violated the human right to private and family life, the human right to found and raise a family, and the human right to non-discrimination on grounds of disability, financial means, or gender. The Court’s conclusions of violations contrary to the American Convention on Human Rights followed from its ruling that, under the Convention, *in vitro* embryos are not “persons,” and do not possess a right to life. Accordingly, the prohibition of IVF to protect embryos constituted a disproportionate and unjustifiable denial of infertile individuals’ human rights. The Court distinguished fertilization from conception, since conception, unlike fertilization, depends on an embryo’s implantation in a woman’s body. Under human rights law, legal protection of an embryo “from conception” is inapplicable between its creation by fertilization and completion of its implantation *in utero*.

## INTRODUCTION

The November 2012 judgment of the Inter-American Court of Human Rights (the Court) on Costa Rica’s constitutional prohibition of *in vitro* fertilization (IVF) (Artavia Murillo *et al*, 2012) addressed alleged human rights violations resulting from the prohibitory ruling of the Constitutional Chamber of the Costa Rican Supreme Court of Justice in 2000 (Judgment No. 2000-02306).

The Constitutional Chamber’s ruling struck down the Ministry of Health Executive Decree of 1995 that authorized IVF and regulated its practice (Executive Decree No. 24029S, 1995). The claims that the 2000 ruling violated human rights of access to IVF were based on provisions of the American Convention on Human Rights (the American Convention). Costa Rica became a party to the Convention in 1970, and accepted the binding jurisdiction of the Court in 1980.

The Constitutional Chamber struck down the Executive Decree on the ground that, because IVF is liable to create human embryos some of which are unavoidably destined to die, the practice affects the “*right to life and dignity of the human being*” (Artavia Murillo *et al*, 2012) (para. 72), which the Executive Branch of government cannot constitutionally regulate. Further, the Constitutional Chamber found a violation of Article 21 of the Costa Rica Constitution, which declares that human life is inviolable, and of Article 4 of the American Convention, on the Right to Life.

Article 4 (1) provides that:

*Every person has the right to have his life respected. This right shall be protected by law and, in general, from the moment of conception. No one shall be arbitrarily deprived of his life*.

The case came to the Court following protracted procedural, legislative and related actions (Artavia Murillo *et al*, 2012) (paras 1-3), but its key issues under the Convention’s legal regime concerned what constitutes a “*person*”, the meaning of “*conception*”, and the significance of the words *“in general”.*

The case attracted widespread international attention, including over 46 amicus curiae (friend of the court) briefs. At the public hearing before the Court, one of the present authors (F.Z-H.) was admitted to present expert opinion on the scientific aspects of IVF, to which the Court made extensive reference.

## HUMAN RIGHTS CLAIMS

The case was initiated in July 2011 by the Inter-American Commission on Human Rights on behalf of 18 named Costa Rican residents, nine couples, whose human rights under the Convention were claimed to be denied by the prohibition of IVF. The claim alleged violation of Articles 11(2), 17(2) and 24 of the Convention.

Article 11(2), concerning the Right to Privacy, provides that “*No one may be the object of arbitrary or abusive interference with his private life, his family, his home…*” Based on the narratives of family distress due to failures of natural conception and pregnancy, the claim advanced was that prohibition of IVF denies infertile couples alternative means of having the children they want, and amounts to violation of their rights to private and family life. Article 17(2), concerning Rights of the Family, provides that:

*The right of men and women of marriageable age to marry and to raise a family shall be recognized, if they meet the conditions required by domestic laws, insofar as such conditions do not affect the principle of non-discrimination established in this Convention*.

Childless couples came before the Court to show how prohibition of IVF denied their only feasible means to pursue their right to seek conception of their genetic progeny. Article 24, on the Right to Equal Protection, provides that *“All persons are equal before the law. Consequently, they are entitled, without discrimination, to equal protection of the law.*” The evidence presented by witnesses showed discrimination on grounds of financial inability to meet the costs of obtaining IVF services in other countries, such as Colombia, Mexico or the USA, and/or on grounds of sex. The personal and expert witnesses reinforced each other’s evidence that the burden of infertility falls more heavily on women than men. Men’s inability to father children has some negative implications for their virility, but they can redeem reputations for manliness in other ways. In contrast, in Latin America, as in many other regions of the world, the culture, reinforced by religious stereotyping, makes the duties of motherhood an indispensable function of adult women’s existence in society, and their main or sole source of esteem within their families and communities. Denial of the potential for parenthood through prohibition of IVF has a disproportionate impact on women. Accordingly, proscribing access to effective, safe methods of overcoming infertility constitutes discrimination against women.

## THE SCIENTIFIC EVIDENCE

The key scientific evidence submitted by the Court-appointed experts related to human reproductive biology and techniques for treatment of infertility, but it also addressed medical and social science evidence of the incidence of infertility and its emotional impact on health and family relationships. In opening by observing that the World Health Organization (WHO) characterizes infertility as a disease (Zegers-Hochschild *et al*, 2009), the evidence showed how the WHO concept of health as a state of “physical, mental and social well-being” (World Health Organization. Constitution) confirms the harmful effect of infertility on the health of couples seeking to be parents, associated with the loss of mental and social well-being due to unfulfilled hopes of parenthood. Inequities in access to IVF, of which those with means can avail themselves by travel to other countries, aggravate infertile couples’ sense of grievance.

The main thrust of the scientific evidence was to inform the judges about techniques of assisted reproduction, especially by varieties of IVF. The description “IVF” has become a generic term for a variety of reproductive techniques. The focus of the evidence addressed the principal objection on the grounds of which the Constitutional Chamber of the Costa Rican Supreme Court found IVF unconstitutional, namely that it may involve wastage of surplus embryos. The scientific evidence did not deny this, but showed such wastage also to be inherent in spontaneous reproduction. Techniques of IVF, being medically controlled and monitored, can calculate with some precision how many embryos are created in vitro, how many are unsuitable for transfer to a woman’s body, how many transferred achieve implantation in utero, how many that implant are subsequently lost, and how many result in pregnancy, gestation and live birth of children. In nature, these processes occur within women’s bodies, and are not amenable to precise calculation or quantification until after embryo implantation has occurred. However, the expert opinion presented to the Court was that:

*The process that generates human life includes embryonic death as part of a natural and necessary process. Of every 10 embryos spontaneously generated in the human species, no more than 2 or 3 are able to survive natural selection and be born as a person. The remaining 7 or 8 embryos die in the female genital tract, generally without the parents’ knowledge* (Artavia Murillo *et al*, 2012) (para. 310).

Whether the rate of loss of embryos in IVF is comparable with or considerably greater than in natural reproduction was contested. One expert claimed that *“embryo mortality is around 30% in natural circumstances and, for IVF… around 90%”* (Artavia Murillo *et al*, 2012) (para. 308), but conceded that early natural embryonic mortality cannot be detected. The Court approached comparative rates of embryonic loss in natural and assisted reproduction less as a quantitative than a qualitative issue in considering whether the Constitutional Chamber’s conclusion that IVF had to be prohibited on grounds of some rate of embryonic loss of life was proportionate and sufficient to justify any violation of infertile couples’ human rights.

In defence of the Constitutional Chamber, the State of Costa Rica argued that the Chamber’s decision favoring protection of embryos by restriction of IVF, in preference to protection of born persons’ freedom to enjoy their human rights, is proportionate, because *“when weighing the harm that the restrictive measure causes to the holder of the freedom, and the benefit that the collectivity obtains from it by protecting society’s most fundamental value, which is the right to life, the State must necessarily incline the balance in favor of the latter”* (Artavia Murillo *et al*, 2012) (para. 270).

The Court accordingly had to decide whether protection of embryos’ alleged right to life justified sacrifice of infertile Costa Rican citizens’ human rights to private and family life, to raise families, to equality and non-discrimination, and, inter alia, to autonomous choice of reproductive treatments (Artavia Murillo *et al*, 2012) (paras 150, 161). Observing that *“the protection of the right to life under this provision [of the Convention] is not absolute”* (Artavia Murillo *et al*, 2012) (para. 264), the Court addressed prioritization of competing rights.

## THE STATUS OF EMBRYOS

The Constitutional Chamber found IVF unconstitutional because Article 21 of the Constitution of Costa Rica provides that *“Human life is inviolable”* and, under the law:

*When the spermatozoid fertilizes the egg that entity becomes a zygote and therefore an embryo …as soon as conception occurs, a person is a person and we are in the presence of a living being, with the right to be protected by the legal system.* (Artavia Murillo *et al*, 2012) (para. 73)

Further, without drawing any distinction between fertilization and conception, the Constitutional Chamber added that, under the American Convention, a human embryo:

*...is a person from the time of conception; hence it cannot be treated as an object…the application of the technique of in vitro fertilization and embryo transfer, as it is currently performed, jeopardizes human life… consequently the [Executive] regulation under consideration is unconstitutional as it violates article 21 of the Constitution and Article 4 of the American Convention on Human Rights. Since the technique violates the right to life…its application cannot be authorized.* (Artavia Murillo *et al*, 2012) (para. 76)

The Court did not consider rights under Costa Rica’s domestic law, but, since Article 7(1) of the national Constitution provides that *“Public treaties, international agreements and concordats duly approved by the Legislative Assembly have a higher authority than the laws upon their enactment”*, its rulings interpreting the American Constitution prevail within Costa Rica.

Noting undefined references to fertilization and conception, the Court addressed how they are to be understood under the American Convention. Drawing upon testimony of its expert witnesses, the Court considered that *“the scientific evidence agrees in making a difference between two complementary and essential moments of embryonic development: fertilization and implantation. The Court observes that it is only after completion of the second moment… that conception can be understood to have occurred”* (Artavia Murillo *et al*, 2012) (para. 186). This accords with the fact that, before implantation, there are no measurable chemical or other markers in the woman’s fluids signalling the presence of an embryo. Only after implantation can an embryo be identified through the woman’s circulation. The Court concluded:

*Thus, the Court considers that the term “conception” cannot be understood as a moment or process exclusive of a woman’s body, given that an embryo has no chance of survival if implantation does not occur. …In addition…when Article 4 of the American Convention was drafted the dictionary of the Real Academia differentiated between the moment of fertilization and the moment of conception, understanding conception as implantation…. When drafting the relevant provisions in the American Convention, the moment of fertilization was not mentioned.* (Artavia Murillo *et al*, 2012) (para. 187)

On extensive review of the history and legal context of the drafting of the American Convention, the Court found an unimplanted embryo to be only human cells or tissues, and concluded that:

*the historic and systematic interpretation of precedents that exist in the inter-American system confirms that it is not admissible to grant the status of person to the embryo* (Artavia Murillo *et al*,2012) (para. 223).

This conclusion related to the Court’s interpretation of the words *“in general”* in Article 4 (1) of the American Convention, which provides that *“Every person has the right to have his life respected. This right shall be protected by law and, in general, from the moment of conception.”* Taking account of scientific data and the jurisprudence of the European Court of Human Rights, on reproductive health including lawful abortion, the Court concluded that:

*...“conception” in the sense of Article 4 (1) occurs at the moment when the embryo becomes implanted in the uterus, which explains why, before this event, Article 4 of the Convention [the Right to Life] would not be applicable. Moreover, it can be concluded from the words “in general” that the protection of the right to life under this provision is not absolute, but rather gradual and incremental according to its development* (Artavia Murillo et al, 2012) (para. 264).

The Court’s ruling that an in vitro embryo is not a person and has no human rights, including to life, is consistent with evolving jurisprudence in Europe and the USA that is coming to address such embryos as a form of property (Dickens & Cook, 2010), disposable by their genetic originators who do not want to use them to become parents (Evans v United Kingdom. European Court of Human Rights Reports, 2007).

## VIOLATION OF HUMAN RIGHTS

In light of the Court’s conclusion on the legal status of in vitro embryos, the Court had to decide whether their protection could proportionately justify any violation of human rights that might follow from the Constitutional Chamber’s absolute prohibition of their creation by IVF. Rejecting the Constitutional Chamber’s perception that *“the application of the technique of in vitro fertilization and embryo transfer… jeopardizes human life”* (Artavia Murillo *et al*, 2012) (para. 76), the Court reviewed the personal case histories of the nine named couples that demonstrated that IVF offered the only means by which their infertility might be overcome to create their children’s lives. The Court accepted that *“one of the purposes of IVF is to contribute to the creation of life”* (Artavia Murillo *et al*, 2012) (para. 311). The Court observed that *“the decision to have [one’s own] biological children using assisted reproduction techniques forms part of the sphere of the right to personal integrity and to private and family life”* (Artavia Murillo *et al*, 2012) (para. 272), protected by Article 11(2) of the American Convention. The Court noted how the prohibition of IVF had caused distress, marital break-down and comparable personal and social ill effects, while, in view of the relatively high rate of embryonic loss in natural reproduction and other reproductive techniques permitted in Costa Rica, *“the protection of the embryo sought by banning IVF has a very limited and moderate scope”* (Artavia Murillo *et al*, 2012) (para. 313). The Court accordingly found a violation of Article 11(2) of the American Convention, since:

*…the Court concludes that the Constitutional Chamber based itself on an absolute protection of the embryo that, by failing to weigh up or take into account the other competing rights, involved an arbitrary and excessive interference in private and family life that makes this interference disproportionate* (Artavia Murillo *et al*,2012) (para. 316).

Similarly, a violation of Article 17(2), concerning Rights of the Family, was found because the prohibition of IVF denied couples dependent on that technique the possibility to raise a family. The Court’s conclusion was that:

*A weighing up of the severity of the limitation of the right to private life and to found a family compared to the importance of the treaty-based protection of prenatal life allows it to be affirmed that the effects on the right to private life, intimacy, reproductive autonomy, access to reproductive health services, and to found a family is severe because, in the practice, these rights are annulled for those persons whose only possible treatment for infertility is IVF* (Artavia Murillo *et al*, 2012) (para. 314).

The Court added that, *“in addition, the interference has a differentiated impact on the victims owing to their situation of disability, gender stereotypes and, for some of the victims, their financial situation.”* The Court did not address direct discrimination in violation of the Right to Equal Protection under Article 24, but addressed the different forms of indirect discrimination disclosed in the evidence of the effects of the prohibition of IVF. It explained that *“the Court will not analyze the presumed violation of the right to equality and non-discrimination under Article 24, but rather in light of Article 1 (1) of the Convention in relation to Articles 11 (2) and 17 thereof”* (Artavia Murillo *et al*, 2012) (para. 285). Article 1(1) is the general duty to respect the rights and freedoms recognized in the Convention without discrimination. Any discrimination in the respect or guarantee of a Convention-based right violates not only the right itself but also Article 1(1).

The Court found that the violations of the human rights to private and family life and to raise a family, resulting from the prohibition of IVF, constituted indirect discrimination in violation of Article 1(1). The Court accepted the WHO characterization of infertility as a disease (Zegers-Hochschild, 2009), and found that legal prohibition of means to overcome its effects discriminates against those the disease disables. Beyond offensive discrimination on grounds of disability, the Court also found discrimination on grounds of financial means, since Costa Ricans were not prohibited from access to IVF services if they could afford the costs of travel to other countries, of which the evidence showed that some had taken advantage.

Expert evidence the Court accepted was particularly concerned with the harmful and discriminatory effect of infertility on women, while the Court agreed with WHO that *“the role and status of women in society should not be defined solely by their reproductive capacity”* (Artavia Murillo *et al*, 2012) (para. 296). It noted evidence of the personal suffering of women due to infertility in their marriages that may be exacerbated by instability in family relationships, domestic violence, stigmatization and ostracism, and observed that:

*The Court considers that the instant case reveals a... situation of the influence of stereotypes, in which the Constitutional Chamber gave absolute prevalence to the protection of the fertilized eggs without considering the situation of disability of some of the women...* (Artavia Murillo *et al*, 2012) (para. 297)

The Court concluded that, while the prohibition of IVF is not expressly addressed at women and appears neutral, it has a disproportionately negative impact on women. The Court was explicit that discriminatory gender stereotypes are incompatible with international human rights law (Cook & Cusack, 2010), and recognized and defined them *“in order to describe the disproportionate impact of the interference caused by the Constitutional Chamber’s judgment”* (Artavia Murillo *et al*, 2012) (para. 302).

## IMPLICATIONS OF THE JUDGMENT

The jurisdiction of the Inter-American Court of Human Rights is very widely if not universally accepted in Latin America, and legal systems of member states of the American Convention are inclined to defer to its rulings. Accordingly, the Court’s judgment concerning IVF in Costa Rica may be expected, in time, to reverberate throughout the region. Constitutional laws and legal systems in the region share several common features, due to a history of colonization and the unifying influence of the Roman Catholic church. Since the decree of Pope Pius IX of 1869 (Luker, 1984), that church has considered deliberate taking of human life to be punishable from conception, and this is reflected in the constitutions and laws of several countries and subordinate jurisdictions, particularly to reinforce prohibition of abortion. For instance, since in 2008 the Supreme Court of Mexico upheld the liberal abortion law of Mexico City, (Acción de Inconstitucionalidad 146/2007, Mexico), most Mexican states have amended their constitutions to ban abortion by requiring protection of embryos “*from conception”.*

The Inter-American Court’s separation of fertilization from conception supports the legal accommodation of IVF in Costa Rica and other countries of the region where it is practiced. In defence of the Constitutional Chamber’s prohibition of IVF, Costa Rica argued that *“scientific evidence...reveals that human life begins with conception, or what is the same, with fertilization”* (Artavia Murillo *et al*, 2012) (para. 167), and that *“the terms ‘conception’ and ‘fertilization’ should be treated as synonyms*” (Artavia Murillo *et al*, 2012) (para. 168). Failure of the legal argument that conception, which is dependent on an embryo’s implantation in utero, includes fertilization, which is not, requires re-examination, for instance of conflating emergency contraception by use of levonorgestrel, which is effective only before completion of conception (Cook *et al*, 2006), with abortion (Hevia, 2012). The Court’s denial that in vitro embryos have a human right to life also eases funding and conduct of embryonic stem cell research. (Dickens & Cook, 2007).

Costa Rica claimed that *“there is no consensus that infertility is, per se, a disease”,* or that it can “be considered a disability,” and that IVF is not a cure because “*it does not constitute a treatment to change the situation that causes a couple or an individual to be infertile*” (Artavia Murillo *et al*, 2012) (para. 271) A cause of infertility is treated, however, when spermatozoa lack fertilizing capacity but IVF or the related procedure of intracytoplasmic sperm injection (ICSI) is applied to insert a sperm into an ovum. When IVF does not treat causes of infertility, it serves to remedy involuntary childlessness, and so promote health, meaning *“physical, mental and social well-being”* (World Health Organization, Constitution) The Court noted expert witness testimony that, consistently with the WHO definition, *“infertility is a disease that has numerous effects on the physical and psychological health of the individual, as well as social consequences, which include unstable marriages, anxiety, depression, social isolation and loss of social status, loss of gender identity, ostracism and abuse”* (Artavia Murillo *et al*, 2012) (para. 288). The Court’s acceptance of infertility as a disease may be invoked to support IVF and comparable funding of reproductive health care through public and private health insurance plans in Latin America, and widely beyond.

Perhaps the least convincing argument by Costa Rica was that the Constitutional Chamber’s judgment was not an absolute prohibition of IVF, but only of all present techniques, because they may involve non-implantation of embryos created in vitro.

The state referred to a law unsuccessfully proposed to the Legislative Assembly in 2010 that would have limited fertilization of eggs in a treatment cycle, and required them all to be transferred at the same time to the woman who produced them, without allowing cryopreservation.

The Court cited the criticism of this proposal by the Pan-American Health Organization (PAHO), based on the *“risks of multiple pregnancies...which also increase the risk of spontaneous abortions, obstetric complications, premature births and neonatal morbidity”* (Artavia Murillo *et al*, 2012) (para. 84). This points to the harms risked to women’s and newborns’ health when laws on human reproduction are modelled on the values of theologians, rather than on evidence-based science and respect for and protection of the human rights of women.

The Court recognizes the diversity by which individuals’ beliefs affect their lives, but establishes the entitlement of all to the human rights of privacy to found families, of ability to raise families according to family preferences, and of non- discrimination on grounds of disability.

The Inter-American Court’s landmark human rights decision, structured upon robust scientific evidence, directs states and governments on the reproductive rights they must provide and protect, and may open new pathways in the defence of women’s rights in the Americas, and potentially beyond.
